# T regulatory cells and the control of alloimmunity: from characterisation to clinical application

**DOI:** 10.1016/j.coi.2010.08.011

**Published:** 2010-10

**Authors:** Joanna Wieckiewicz, Ryoichi Goto, Kathryn J Wood

**Affiliations:** Transplant Research Immunology Group, Nuffield Department of Surgical Sciences, University of Oxford, Oxford, UK

## Abstract

T regulatory cells (Treg) play an important role in the induction and maintenance of immunological tolerance. Recent findings in experimental transplant models combined with the development of functional reporter mice have opened new avenues to study Treg biology and their therapeutic potential. In particular, recent advances in understanding Treg function and lineage stability revealed unexpected plasticity of this lineage. Nevertheless, pre-clinical and pilot clinical trials using Treg cells as cellular therapies have been initiated suggesting the safety and feasibility of such treatment.

## Introduction

The appropriate balance between effector and T regulatory cells (Treg) is indispensable for the maintenance of self-tolerance and of functional immune responses *in vivo*. In the transplant setting, numerous findings during the last 25 years have demonstrated the importance and therapeutic potential of Treg in the active control of rejection responses. Although the existence of various cell populations with regulatory/suppressive activity, such as IL-10 secreting Tr-1 cells, CD28^−^CD8^+^ cells or B regulatory cells, has been demonstrated, in this review we will focus on classical CD25^+^CD4^+^ Treg cells.

Treg mediate suppressive effects by several mechanisms including anti-inflammatory cytokines (IL-10, TGF-β and IL-35), direct cytotoxic effect (granzyme B and galectin-1), metabolic disruption (adenosine production and IL-2 deprivation) and modulation of dendritic cell function (CTLA-4, LAG3 and IDO induction) as discussed in [1]. Numerous regulatory mechanisms described to date suggest that multiple, redundant mechanisms are required for optimal Treg function *in vivo*.

Treg can be divided into two populations: thymic-derived naturally occurring CD25^+^CD4^+^ cells (nTreg) [2] and induced or adaptive Treg (iTreg) that are either differentiated from CD25^−^CD4^+^ nonregulatory cells or expanded from CD25^+^CD4^+^ cells in response to the antigen [3]. These cells differ in origin, antigen experience, methylation patterns of the key transcription factor FoxP3 and suppressive mechanisms. Recently, the differential expression of transcription factor Helios has been attributed to thymic-derived nTregs and proposed as a marker to differentiate between nTregs and iTregs [4^••^]. Both nTreg and iTreg have been demonstrated to play an important role in transplant tolerance.

In this review, we explore recent advances in understanding Treg function and lineage stability and its impact on tolerance induction protocols. Next, we focus on the current attempts to expand human Treg for clinical application as a cellular therapy in organ and cell transplantation. Finally, we discuss the arising or potential issues relating to the therapeutic application of Treg and proposed solutions.

## FoxP3 expression in Treg

Expression of the transcription factor FoxP3 is essential for the development and function of Treg [5,6]. It was demonstrated that ectopic expression of Foxp3 in conventional T cells was sufficient to confer suppressive activity, repress IL-2 and IFNγ production and upregulate Treg-associated molecules such as CTLA-4 and GITR [2,7]. Furthermore, expression of FoxP3 in mature Treg is necessary for the maintenance of Treg-specific transcription profile and of Treg function [8].

### Epigenetic regulation of FoxP3 expression

Several epigenetic markers, such as histone acetylation and methylation, and cytosine residue methylation in CpG dinucleotides, have been reported at the Foxp3 locus [9^•^]. In particular, a unique CpG-rich island within an evolutionarily conserved region upstream of exon 1, named TSDR (Treg-specific demethylation region), was demonstrated to be unmethylated in natural Treg but heavily methylated in other CD4+ T cells [10^•^,11]. Demethylation of these CpG sites resulted in strong and stable induction of FoxP3. In human, upon *in vitro* expansion of Treg, CpG methylation increased correlating with loss of FoxP3 expression and emergence of pro-inflammatory cytokines [12^•^]. Interestingly, CD45RA^+^FoxP3^+^ naïve Treg showed no increase in CpG methylation after 3-week culture, whereas CD45RA^−^FoxP3^+^ memory-like Treg from the same donors lost CpG demethylation status and converted into non-Treg cells. Recent advances in our understanding of the complex regulation of FoxP3 expression have led to new methods of analysing Treg based on quantitative DNA methylation analysis of FoxP3 locus [13^•^], which may add a useful test for quality assessment of *ex vivo* manipulated Treg cells.

### Treg lineage stability

FoxP3 epigenetic analysis and the development of functional reporter mice questioned the dogma of natural Treg lineage stability. An elegant study by Zhou *et al.* examined the stability of Treg cells by tracing cells that induced and downregulated FoxP3 during their life span [14^••^]. The authors found that cells that at some point expressed FoxP3 and lost its expression shared their TCR repertoire both with FoxP3^+^ Treg cells and with conventional T cells suggesting that they originated from both nTreg and iTreg. These ‘ex-Treg’ had an activated-memory phenotype and produced pro-inflammatory cytokines. Notably, an autoimmune microenvironment favoured loss of FoxP3, and ‘ex-Treg’ cells from diabetic mice were able to transfer diabetes [14^••^]. Notably for the transplant setting, it was also demonstrated that some peripheral FoxP3^+^CD4^+^ cells lose their FoxP3 expression and start producing IFNγ and IL-17 after transfer to a lymphopenic host [15^•^].

## Cellular therapy with Treg

### Mouse pre-clinical models

Many strategies exist for the *in vivo* or *ex vivo* generation and/or expansion of Treg. The most common *in vivo* approaches are based on the fact that exposure to antigen increases Treg frequency and/or potency by either expanding naturally occurring Treg or inducing the generation of adaptive Treg from cells that do not originally possess regulatory activity [16^•^]. Generation of Treg can be achieved by attenuation of activating signals during antigen presentation. In the mouse, donor-specific transfusion (DST) combined with a nondepleting anti-CD4 antibody generates CD25^+^CD4^+^ cells able to prevent skin graft rejection [17]. Moreover *in vitro* culture of mouse CD4^+^ or CD25^−^CD4^+^cells in the presence of alloantigen and anti-CD4 antibody results in the enrichment of CD62L^+^CD25^+^ cells effective in controlling graft survival [18]. Interestingly, conditioning of CD4^+^ cells in the presence of interferon-γ (IFN-γ) and immature DC can also generate FoxP3^+^ cells that are able to protect both skin and islet transplants from rejection [19^•^,20]. Notably, alloantigen-reactive Treg from *in vivo* tolerised mice demonstrate increased levels of IFN-γ production transiently after antigen-specific reactivation through T cell receptor [21^•^]. *In vivo*, IFNγ produced locally where the Treg are present, the draining lymph nodes and the graft [22^•^], creates a microenvironment that influences the function of other cells in the vicinity, including the Treg themselves where evidence for the activation of IFNγ signalling pathways has been reported [21^•^].

Another approach to enrich Treg *in vivo* is to create Treg-favouring conditions. In the transplantation setting, patients are treated with diverse immunosuppressive drug combinations, which may have a different impact on Treg. It was demonstrated that calcineurin inhibitors (CNI), especially cyclosporine A, are detrimental to Treg, whereas the mTOR inhibitor rapamycin was shown to be beneficial for Treg both in terms of *in vivo* generation and function in mouse models [23] and in *in vitro* cultures of human Treg [24]. It was recently demonstrated that adoptive transfer of a low number of alloantigen-specific Treg under a cover of low dose of rapamycin induced long-term survival of heart transplant in unmanipulated host, an outcome otherwise difficult to obtain [25]. Interestingly, in terms of alloantigen-specificity of Treg two recent papers have independently demonstrated that regulatory cells specific for both directly (by donor APC) and indirectly (by host APC) presented alloantigens prolonged graft survival with substantially greater efficacy than Treg with only direct anti-donor specificity [26^•^,27^•^]. Noteworthy, successful attempt to achieve long-term acceptance of islet allografts without immunosuppression was demonstrated by Webster *et al.* who *in vivo* expanded Treg by injecting mice with IL-2/anti-IL-2 monoclonal antibody complexes [28^•^].

### Human Treg

Human Treg are currently less well characterised and understood than mouse Treg, so a thorough understanding of their biology is vital before clinical applications can be initiated. It is also important to highlight that there are substantial differences between human and mouse Treg; most notably the differences in FoxP3 expression between mouse and human. In human, FoxP3 is also expressed by activated nonregulatory T cells as well as by Treg, and activated nonregulatory cells also upregulate CD25 expression. Thus not all CD25^+^FOXP3^+^CD4^+^ will be genuine Treg and therefore isolation strategies based on CD25^hi/+^CD4^+^ are likely to be imperfect. Other markers are therefore needed to enrich Treg from human peripheral blood mononuclear cells. Recently, it has been demonstrated that CD127^lo^CD25^+^CD4^+^ T cells are characterised by a higher percentage of FoxP3^+^ cells with a more pronounced suppressive capacity [29,30]. Expansion of CD127^lo^CD25^+^CD4^+^ cells resulted in high yield of regulatory cells which maintained high FoxP3 expression [31^••^]. Importantly, as we recently demonstrated in a clinically relevant humanised model of transplant arteriosclerosis, *ex vivo* expanded CD25^hi^CD4^+^ and CD127^lo^CD25^+^CD4^+^ Treg cells have been very effective in inhibiting vasculopathy, with CD127^lo^CD25^+^CD4^+^ cells being five times more efficient than conventional Treg [32^••^]. Interestingly, another study subdivided Treg into two subtypes: resting naïve CD25^+^CD45RA^+^FoxP3^lo^ and activated CD25^hi^CD45RA^−^FoxP3^hi^ regulatory cells. A third population of FoxP3^+^ cells phenotyped as CD25^+^CD45RA^−^FoxP3^lo^ was demonstrated to consist of cytokine-secreting, non-suppressive cells [33^•^]. Notably, whereas both regulatory subpopulations were highly suppressive *in vitro*, only resting Treg were able to proliferate *in vitro* and *in vivo*, converting into activated CD45RA negative Treg.

**Figure 1:**
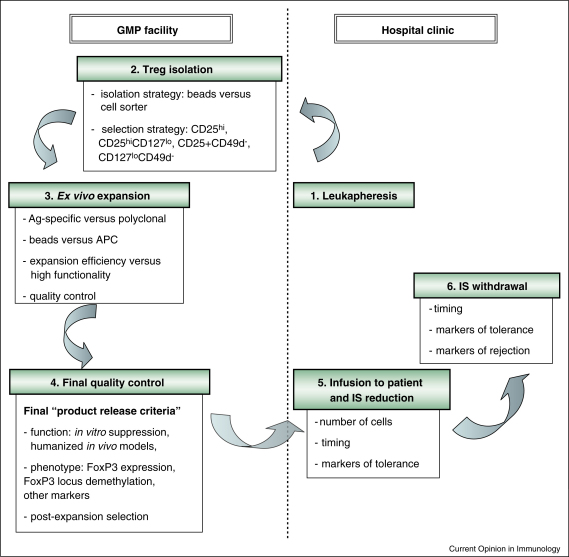
Steps in preparation and clinical application of Treg cells. In transparent boxes questions awaiting answers in the process of developing Treg cellular therapy. IS—immunosuppression; GMP—good manufacturing practices.

A number of different strategies for the isolation/enrichment of human Treg have been described in the literature, but to date there is no consensus as to which strategy produces the optimal population for use in cell therapy applications. The critical steps and the questions awaiting answers in the process of developing clinically approved Treg cellular therapy are outlined in Figure 1.

### Clinical application of human Treg

One of the obstacles in the implementation of clinical protocols using Treg is their low frequency in the peripheral blood leading to the need for *ex vivo* multiplication of the cells prior to their use *in vivo*. The most commonly used expansion protocol at present is based on stimulation by anti-CD3/anti-CD28 beads in the presence of high doses of recombinant IL-2, supplemented in some protocols with rapamycin. This protocol results in the efficient expansion of polyclonal Treg, generating sufficient numbers of cells for cellular therapy [34^•^]. However, the expansion is antigen non-specific without any enrichment step for the cells of interest. More appealing for clinical application, is the concept of expanding or generating antigen-specific Treg, in the setting of transplantation, donor alloantigen-reactive Treg. Interestingly, human Treg expanded with allogeneic PBMC, were found to be more suppressive *in vitro* than polyclonaly-driven cells and, surprisingly, expanded at a similar rate as polyclonaly-stimulated cells [35].

### Clinical trials

There are several ongoing clinical trials with the application of Treg cellular therapy. To date regulatory cells have been adoptively transferred into haematopoietic stem cell transplant (HSCT) recipients in Germany, USA and Italy [36^•^]. In one of such trials, led by Matthias Edinger from Regensburg, Germany, freshly isolated bead-selected donor Treg were infused into HSCT recipients. So far nine patients have been included in the study with no side effects (M. Edinger, personal communication). In a study led by Massimo Martelli, 22 patients were inoculated with freshly isolated donor Treg followed by infusion of haematopoietic stem cells and donor conventional T cells. This strategy improved immune recovery after HSCT without causing graft versus host disease (GVHD) (Di Ianni *et al.*, 51st ASH Annual Meeting and Exposition, New Orleans, LA, December 2009). Another clinical trial in the University of Minnesota, USA, led by Bruce Blazar and co-workers, used *ex vivo* expanded bead-enriched, third-party, partially HLA-matched Treg cells from umbilical cord blood (UCB). These UCB Treg cells were administered into myeloablated or nonmyeloablated recipients of two unrelated UCB units [36^•^].

Whereas aforementioned studies utilised Treg to prevent GVHD, Trzonkowski *et al.* [34^•^] have reported their findings from two patients with GVHD who were treated with anti-CD3/anti-CD28 bead expanded CD25^+^CD127^lo^ Treg. One of the patients, with chronic GVHD, responded to the therapy with alleviation of the symptoms and reduction of immunosuppression. However, in the case of the second patient with acute, grade IV GVHD, only transient improvement was observed. Notably, in the case of the patient treated successfully with cellular Treg therapy, only 1 × 10^5^ Treg cells/kg body weight was sufficient to achieve clinical improvement. Published reports and unpublished results from other ongoing studies indicate that Treg cellular therapy is proving to be effective in clinical situations. Currently, further clinical studies are being planned to apply Treg therapy in solid organ transplantation.

## Questions arising

### IL-17 production by FoxP3+ cells and Treg lineage stability

The ability of *in vitro* expanded Treg to convert into cytokine producing, nonregulatory cells upon prolonged TCR stimulation [12^•^] has led to concerns about the efficacy and safety of *ex vivo* Treg expansion protocols. Importantly, it was demonstrated that a proportion of circulating Treg have the capacity to secrete IL-17 and express RORγt [37,38]. These IL-17 producing cells are of memory phenotype and express CCR6, a marker associated with Th17 cells. Interestingly, some authors have shown that IL-17 producing CCR6^+^ Treg are as equally suppressive as CCR6^−^ Treg [37,38], whereas others demonstrated a loss of suppressive function in FoxP3^+^IL-17^+^ clones after strong TCR stimulation in the presence of APC [39]. Although the function and regulatory properties of these cells *in vivo* are still debatable, the possibility that they may elicit unwanted responses when transferred to patients cannot be excluded. However, several markers of ‘true’ Treg have been described such as IL-1R2, LAP and GARP that may allow additional post-expansion purification steps to be introduced into clinical protocols to reduce the risk of introducing cells that do not have regulatory function [40^•^,41^•^]. Alternatively, the use of a pure, conversion resistant Treg subpopulation (CD25^+^CD45RA^+^CD127^lo^) as a starting population for expansion [12^•^] would also reduce any safety concerns.

Another way to prevent conversion into Th17 cells may be to use pharmacologic intervention during *ex vivo* culture. Recently, retinoic acid (RA) has been described as inhibiting IL-17 polarisation and to promote FoxP3 expression [42]. Another pharmacologic agent, already in use, as discussed above, is rapamycin which was demonstrated to improve suppressive activity and FoxP3 expression in *ex vivo* expanded human Treg, especially when isolated with magnetic beads [24].

Introduction of additional factors to the *in vitro* Treg cultures that maintain FoxP3 expression in *ex vivo* manipulated cells appears to be an attractive way of ensuring their effectiveness and safety for the patient. Additionally, obtaining cells with higher per cell activity would potentially allow a significant reduction in the number of Treg required for each clinical application.

### Cancer and infection immunity

One of the concerns regarding the application of Treg in transplant recipients is the possibility of inhibition of anti-tumour and antiviral immunity. Theoretically, infusion with large numbers of potent suppressor cells may present a serious obstacle to the induction of effective immune responses towards infectious pathogens and a reduction in immune surveillance against tumour cells. As is often the case with the immune system, reality may not fit with theoretical predictions. For example, *in vivo* ablation of Treg was recently demonstrated to lead to accelerated fatal infection during mucosal herpes simplex virus infection, suggesting that in some situations Treg facilitate early protective responses to local viral infection [43]. Notably, it has also been demonstrated in mice that antigen-specific, *in vivo* induced Treg which were able to induce tolerance in primary and secondary allograft recipients did not affect anti-influenza response even when bystander regulation was deliberately induced [44].

Numerous studies suggest that the accumulation of Treg at tumour sites may affect anti-tumour immunity, therefore infusion of substantial numbers of Treg may particularly influence the response towards already existing early tumours. Notably, high numbers of Treg in the blood have been recently associated with increased risk of new tumour development in kidney transplant recipients with non-malignant squamous cell carcinoma [45]. On the other hand, immunosuppressive agents currently being used are themselves associated with increased risk of cancer [46]. Therefore, careful screening and monitoring of transplant recipients eligible for Treg cellular therapy should be performed before and after infusion in any pilot clinical study.

## Conclusion

Recent progress in understanding Treg biology and the development of experimental mouse models has highlighted potential avenues in the translation of research-based knowledge to the clinic. Insights into the biological role of FoxP3, the effects of immunosuppression on Treg and new protocols to expand or induce Treg provide a knowledge base for developing clinical strategies to achieve long-term graft survival without life-long immunosuppression.

## Conflict of interest

Authors declare no conflict of interest.

## References and recommended reading

Papers of particular interest, published within the period of review, have been highlighted as:

• of special interest
•• of outstanding interest
